# Mesenchymal Stem Cell-Exosomal miR-99a Attenuate Silica-Induced Lung Fibrosis by Inhibiting Pulmonary Fibroblast Transdifferentiation

**DOI:** 10.3390/ijms252312626

**Published:** 2024-11-25

**Authors:** Xiaohui Hao, Peiyuan Li, Yudi Wang, Qinxin Zhang, Fang Yang

**Affiliations:** 1 School of Public Health, North China University of Science and Technology, Tangshan 063210, China; haoxiaohui@ncst.edu.cn (X.H.); pyli2018@163.com (P.L.); wangyudi011215@163.com (Y.W.); a15033963732@163.com (Q.Z.); 2 Hebei Key Laboratory of Organ Fibrosis, North China University of Science and Technology, Tangshan 063210, China; 3 Hebei Coordinated Innovation Center of Occupational Health and Safety, North China University of Science and Technology, Tangshan 063210, China

**Keywords:** silicosis, MSC-exosomes, miR-99a-5p, fibroblast transdifferentiation, fibroblast growth factor receptor3, pulmonary fibrosis

## Abstract

Silicosis is one of the most prevalent and fatal occupational diseases worldwide, with unsatisfactory clinical outcomes. This study aimed to investigate the therapeutic effect and related molecular mechanisms of how mesenchymal stem cell (MSC)-secreted exosomes alleviate SiO_2_-induced pulmonary fibrosis. miR-99a-5p was significantly downregulated in silicosis models via high-throughput miRNA screening, and was overlapped with miRNAs in exosomes from MSCs. miR-99a-5p was significantly downregulated in the lung of a mice silicosis model and in TGFβ1-induced NIH-3T3 cells. In contrast, fibroblast growth factor receptor 3 (*FGFR3*), a direct target gene of miR-99a-5p, was upregulated in vitro and in vivo. Furthermore, we demonstrated that MSC-derived exosomes deliver enriched miR-99a-5p to target cells and inhibit TGF-β1-induced fibroblast transdifferentiation to reduce collagen protein production. Similarly, in a silicosis mouse model, MSC-derived exosome treatment through the tail veins of the mice counteracted the upregulation of fibrosis-related proteins and collagen deposition in the lung of the mice. By constructing exosomal therapeutic cell models with different miR-99a expressions, we further demonstrated that miR-99a-5p might attenuate pulmonary fibrosis by regulating target protein FGFR3 and downstream mitogen-activated protein kinase (MAPK) signalling pathways. Our study demonstrated that MSC-derived exosomes ameliorate SiO_2_-induced pulmonary fibrosis by inhibiting fibroblast transdifferentiation and represent an attractive method of pulmonary fibrosis treatment.

## 1. Introduction

Silicosis is caused by the long-term inhalation of dust with a high level of free silica in the occupational environment, and has historically been common among miners [1,2]. However, its incidence is increasing worldwide due to the development of emerging industries such as stone processing, glass manufacturing, and electronic communication [3]. The pathological process of silicosis mainly consists of persistent chronic inflammation and fibrosis, culminating in respiratory failure and death [4,5]. Lung fibroblast transdifferentiation is crucial for the pathological progression of pulmonary fibrosis [6]. Fibroblasts transdifferentiate into myofibroblasts and secrete excessive extracellular matrices (ECM), a hallmark of silicosis [7]. Excessive ECM accumulation results in mechanical cues that promote fibroblast activation, which leads to pulmonary fibrosis. Therefore, controlling lung fibroblast transdifferentiation is crucial for the development of promising therapeutic approaches [8]. Transforming growth factor (TGF)-β1 is a recognised pro-fibrotic cytokine [9]; we have also constructed an in vitro model of silicosis from TGF-β1-treated NIH-3T3 mouse fibroblasts for further study.

Emerging evidence suggests that mesenchymal stem cells (MSCs) can be used to treat various lung diseases, including pulmonary fibrosis [10,11]. Furthermore, multiple preclinical studies have demonstrated improvements in several pulmonary diseases following MSCs administration, including idiopathic fibrosis and chronic obstructive pulmonary disease [12,13]. MSCs exert therapeutic effects mainly through their secretomes, of which, exosomes are an important component [14]. Exosomes are membrane-bound vesicles produced by multiple cells, being released into the culture medium in vitro [15]. Exosomes range from 30 to 150 nanometres in diameter and are widely involved in intercellular communication and the loading of bioactive substances such as mRNA, miRNA, and proteins [16]. Therefore, they are considered natural nanocarriers with promise for clinical application [17]. MSC-derived exosomes have been shown to attenuate SiO_2_-induced pulmonary fibrosis [18]. However, the mechanisms underlying the effect remain unclear.

miRNAs are non-coding single-stranded RNA molecules that participate in gene silencing via mRNA degradation or the inhibition of translation [19]. The dysregulation of miRNA expression has been linked to various lung diseases, including SiO_2_-induced pulmonary fibrosis [20,21]. MSC-derived exosomes are enriched in miRNAs and delivered to target cells through exocytosis where they may elicit therapeutic effects [16,22]. Hence, it is important to elucidate the connections between exosome-derived miRNAs from MSCs and the regulatory mechanisms of the treatment for silicosis. Our previous results suggested that miR-99a (miR-99a-5p and miR-99a-3p) was significantly downregulated in in vivo silicosis models via high-throughput microRNA screening [20]. Recently, fibroblast growth factor3 (FGFR3), one of the most important targets of miR-99a-5p, has been receiving more attention in the formation of fibrosis [23]. Nevertheless, the regulatory mechanism of FGFR3 in silicosis is still not clear. In our study, we attempt to identify whether the downregulation of miR-99a-5p elevates FGFR3 to promote fibroblasts transdifferentiation in silicosis. Here, we wish to assess the effects of MSC-exosomes in a mouse model of silicosis and a cell model and explore the underlying mechanisms.

## 2. Results

### 2.1. miR-99a-5p Was Significantly Downregulated and FGFR3 Was Upregulated in Pulmonary Fibrosis

**Figure 1:**
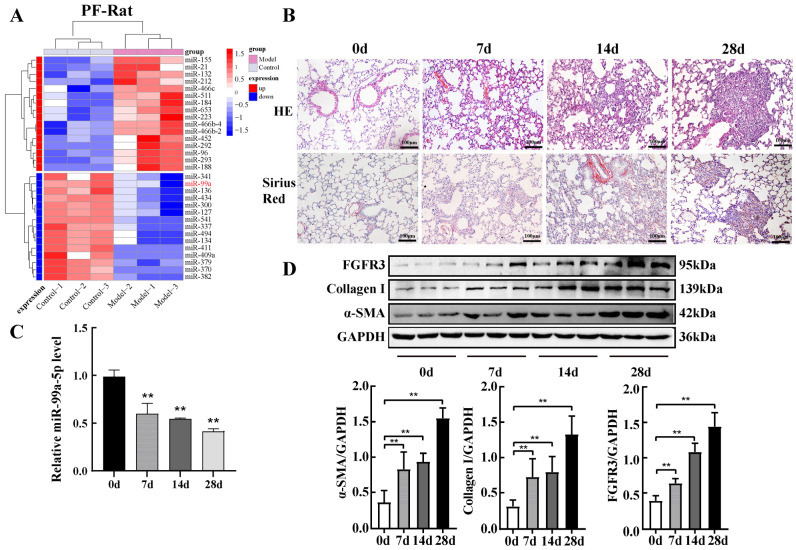
miR-99a-5p was significantly downregulated and FGFR3 was upregulated in pulmonary fibrosis. (**A**) Heatmap representing the differentially expressed miRNAs (red indicates high expression; blue indicates low expression) and sample clustering. (**B**) Morphological changes and collagen deposition in mouse lung tissues were observed via haematoxylin and eosin (HE) and Sirius red staining. (**C**) Expression levels of miR-99a-5p in mouse lung tissue were measured using qRT-PCR. (**D**) Expression levels of FGFR3, α-SMA, and Collagen I protein in lung tissue were measured by Western blot analysis. ** *p* < 0.01 vs. control group (0 d), n = 6.

Previous studies have shown that miRNA dysregulation is closely associated with SiO_2_-induced pulmonary fibrosis. Our previous high-throughput miRNA screen in rats with silicosis showed significant differences in the expression of 31 miRNAs, including 16 and 15 miRNAs with high and low expression, respectively (Figure 1A). Among them, the expression of miR-99a-5p was significantly downregulated in the lung of the animal model. Next, we constructed a mouse model of silicosis to validate the expression level of miR-99a-5p in SiO_2_-induced pulmonary fibrosis. A histopathological examination showed a thickening of the lung interstitium with considerable inflammatory cells infiltrating after 14 d of silica exposure. After 28 d of exposure, massive collagen fibres were deposited, with typical diffuse fibrosis and silica nodules in the lungs of the model mice (Figure 1B). Subsequently, we detected the expression of miR-99a-5p in a mouse model of silicosis using qPCR. The results showed a continuous decrease in miR-99a-5p expression with the increasing duration of SiO_2_ treatment, with the lowest expression observed at 28 d (Figure 1C). Finally, Western blotting analysis indicated that the expression of FGFR3, α-SMA, and Collagen I protein, markers of fibroblast to myofibroblast transdifferentiation, continually rose with increasing silica exposure time (Figure 1D).

### 2.2. miR-99a-5p Was Significantly Downregulated and FGFR3 Was Upregulated in TGF-β1-Induced Pulmonary Fibroblast Transdifferentiation

**Figure 2:**
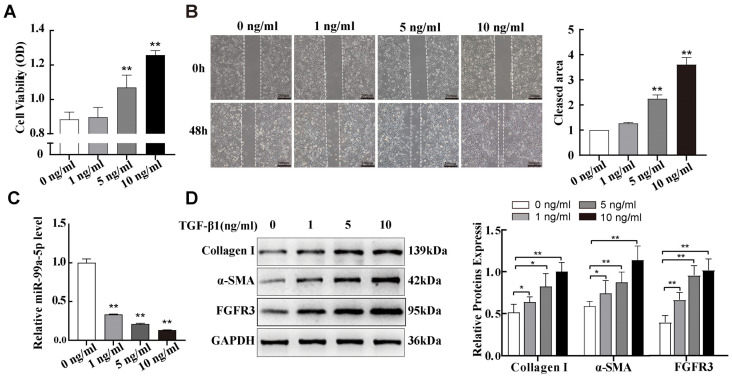
miR-99a-5p was significantly downregulated and FGFR3 was upregulated in TGF-β1-induced pulmonary fibroblast transdifferentiation. (**A**,**B**) MTS cell proliferation and wound healing assays of NIH-3T3 cells treated with different concentrations of TGF-β1. (**C**) Expression of miR-99a-5p in NIH-3T3 cells treated with different concentrations of TGF-β1 was assessed via qPCR. (**D**) Western blotting of α-SMA, Collagen I, and FGFR3 protein levels in NIH-3T3 cells treated with different concentrations of TGF-β1. * *p* < 0.05, ** *p*< 0.01 vs. control group (0 ng/mL), n = 3.

Previous studies have shown that TGF-β1, a crucial pro-fibrotic factor, promotes fibroblast activation to myofibroblast [24]. In this study, we used NIH-3T3 cells, a mouse embryonic fibroblasts cells line, for in vitro studies. We first observed the proliferation and migration abilities of NIH-3T3 cells using MTS cell proliferation assays and wound-healing assays after 48 h of exposure to various concentrations of TGF-β1. Compared with the control group (0 ng/mL), the proliferation and migration abilities of NIH-3T3 cells increased with increasing TGF-β1 concentrations (Figure 2A,B). Next, similar to the silicotic model results, the levels of miR-99a-5p decreased in TGF-β1-treated NIH-3T3 cells with increasing TGF-β1 concentrations (Figure 2C). Then, Western blot data indicated that expressions of the cellular transdifferentiation marker α-SMA and fibrosis marker Collagen Ⅰ were also significantly elevated in a TGF-β1 concentration-dependent manner; FGFR3 protein expression levels also progressively increased in the same way (Figure 2D). Based on these results, subsequent experiments were performed using 10 ng/mL of TGF-β1 as a positive control.

### 2.3. In Vitro Co-Culture with MSCs Reduced TGF-β1-Induced Pulmonary Fibroblast Transdifferentiation

**Figure 3:**
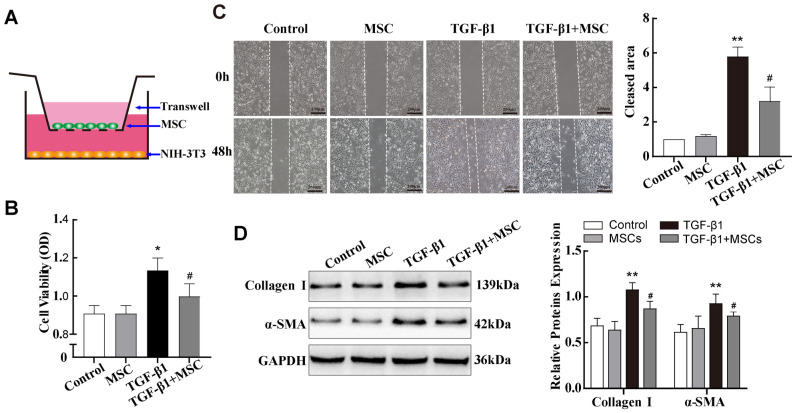
Co-culture with MSCs reduced TGF-β1-induced pulmonary fibroblast transdifferentiation in vitro. (**A**) Schematic design of the transwell experiments. (**B**,**C**) MTS cell proliferation and wound-healing assays were used to evaluate the viability and migration abilities of TGF-β1-treated NIH-3T3 cells co-cultured with MSCs. (**D**) Expression levels of Collagen I and α-SMA in NIH-3T3 cells in different treatment groups were assessed by Western blotting. * *p* < 0.05, ** *p*< 0.01 vs. control group, ^#^ *p* < 0.05 vs. TGF-β1 group, n = 3.

Studies have shown that MSCs contribute to reducing SiO_2_-induced pulmonary fibrosis [11,25]. However, the molecular mechanisms and regulatory networks underlying this process remain unclear. Based on the above results, subsequent experiments were performed using 10 ng/mL of TGF-β1 as a positive control. First, mouse adipose-derived MSCs were isolated from epididymal white adipose of 6-week-old C57BL/6J mice and immortalised by transformation with SV40 virus large T antigens. Then, we also constructed a co-culture system to evaluate the therapeutic effect of MSCs on TGF-β1-treated NIH-3T3 cells (Figure 3A). MTS cell proliferation and migration assays showed that co-culture with MSCs significantly inhibited the proliferation and migration ability of TGF-β1-treated NIH-3T3 cells (Figure 3B,C). Notably, Western blot data showed that α-SMA and Collagen I expressions in NIH-3T3 cells were significantly decreased after co-culture with MSCs (Figure 3D). These results indicated that MSCs may attenuate lung fibrosis by inhibiting fibroblast transdifferentiation.

### 2.4. MSC-Exosomes Attenuated TGF-β1-Induced Pulmonary Fibroblast Transdifferentiation

**Figure 4:**
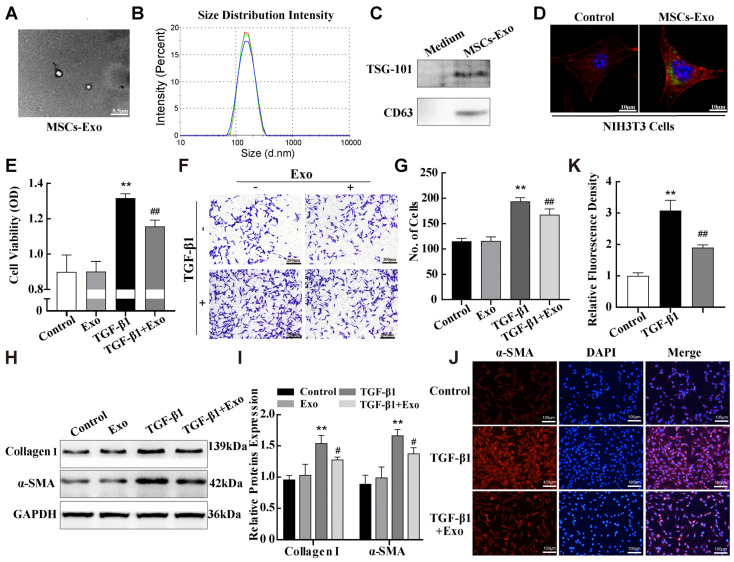
MSC-exosomes attenuated TGF-β1-induced pulmonary fibroblast transdifferentiation. (**A**) Morphology of MSC-exosomes was observed via transmission electron microscopy. (**B**) The particle sizes of exosomes were measured using nanosight particle tracking analysis. Red, green and blue curves represent n = 3 independent preparations. (**C**) Western blotting assays were used to detect exosome surface markers. (**D**) DiO-staining was used to confirm that exosomes could be absorbed by target cells. Green, Dio labelled exosome; red, TRITC-phalloidin labelled actin in NIH3T3; blue, DAPI labelled nucleus in NIH3T3. (**E**–**G**) MTS cell proliferation and transwell assays were performed to evaluate NIH-3T3 cell viability and migration abilities. (**H**–**K**) Western blotting and immunofluorescence assays were performed to examine the expression of α-SMA and Collagen I in NIH-3T3 cells. Data are presented as the means ± SD of values from experiments performed at least in triplicate. ** *p* < 0.01 vs. control group; ^#^ *p* < 0.05, ^##^ *p* < 0.01 vs. TGF-β1 group, n = 3.

It is now widely believed that MSCs exert their therapeutic effect mainly by paracrine secretion, and exosomes are the primary vehicles for MSC function because of their ability to transport large amounts of nucleic acids and proteins [26]. Based on the above data, we hypothesised that the alleviating effect of MSCs in the fibrosis cell model might be accomplished through exosomes. We isolated and purified exosomes from the conditioned media of mouse adipose MSCs via ultracentrifugation using standard exosome isolation methods. Transmission electron microscopy and nanosight particle tracking analysis were performed to identify the structures and sizes of the isolated particles. As shown in Figure 4A,B, the particles had a typical round exosome morphology and ranged from 70 to 160 nm in diameter. In addition, Western blotting revealed the presence of surface markers TSG101 and CD63 on the isolated exosomes (Figure 4C). Collectively, these results indicate that the isolated particles are exosomes. We then assessed whether MSC-derived exosomes were absorbed by NIH-3T3 cells. Green DiO-labelled exosomes were incubated with NIH-3T3 cells for 4 h, and the cells were stained with red TRITC-phalloidin to visualise cell structures. Confocal microscopy showed that DiO green fluorescence appeared inside the NIH-3T3 cells (Figure 4D), indicating that NIH-3T3 cells adequately absorbed the exosomes.

To evaluate the effects of MSC-exosomes on pulmonary fibrosis, we constructed in vivo and in vitro exosome therapy models. MTS assay was performed to optimize the treatment concentration of MSC-exosomes to inhibit the proliferation of NIH-3T3 cells induced by 10 ng/mL TGF-β1. An optimal MSC-exosomes protein concentration of 50 μg/mL was used for all subsequent experiments in vitro (Figure S1). NIH-3T3 cells were treated with 50 μg/mL MSC-exosomes for 24 h after 10ng/mL TGF-β1 stimulation, and MTS cell proliferation and transwell assays were performed to examine the proliferation and migration abilities of NIH-3T3 cells. The results showed that exosome treatment reduced the proliferation and migration of NIH-3T3 cells compared with that in the TGF-β1 treatment group (Figure 4E–G). Importantly, compared with the TGF-β1 group, exosome treatment also significantly decreased the expression of fibrosis markers α-SMA and Collagen I in TGF-β1-stimulated NIH-3T3 cells (Figure 4H,I). Similarly, the α-SMA protein labeled with red fluorescent in NIH-3T3 cells was weakened in the exosome treatment group, compared with the TGF-β1 group (Figure 4J,K). These results demonstrate MSC-exosome treatment inhibits the TGF-β1-induced transdifferentiation of fibroblasts in vitro.

### 2.5. miR-99a-5p Transmitted by MSC-Derived Exosomes Inhibited TGF-β1-Induced Pulmonary Fibroblast Transdifferentiation by Directly Targeting FGFR3

**Figure 5:**
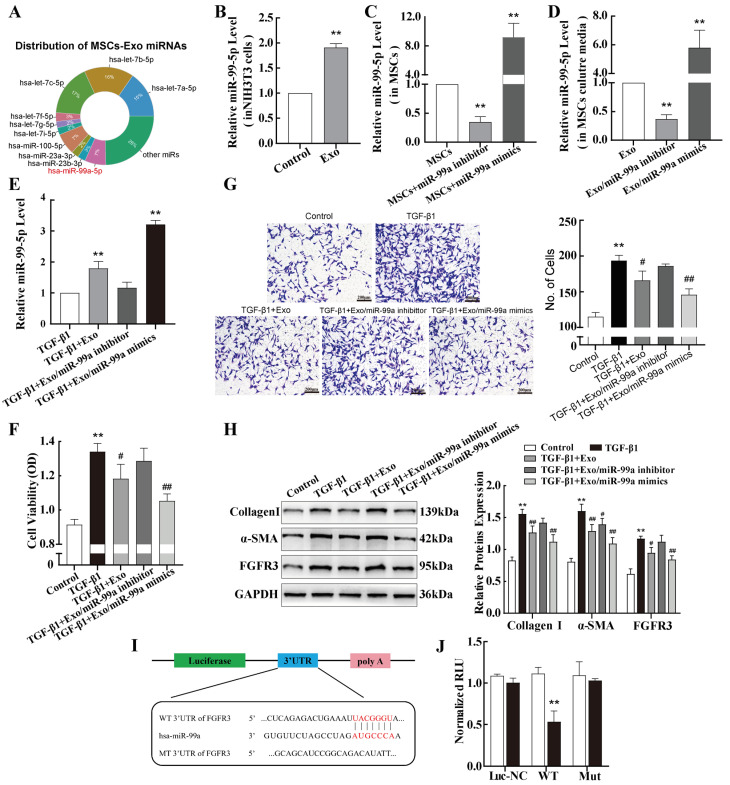
miR-99a-5p transmitted by MSC-derived exosomes inhibits TGF-β1-induced pulmonary fibroblast transdifferentiation by directly targeting FGFR3. (**A**) Distribution of the top-10 miRNAs in MSC-exosomes. (**B**) qPCR was used to determine miR-99a-5p levels in NIH-3T3 cells treated with MSC-exosomes. (**C**,**D**) Levels of miR-99a-5p were determined via qPCR in MSCs transfected with miR-99a-5p mimics or inhibitors and their secreted exosomes. (**E**) qPCR was used to determine miR-99a-5p levels in exosome-treated NIH-3T3 cells. (**F**,**G**) MTS cell proliferation and transwell assays were performed to evaluate the viability and migration abilities of NIH-3T3 cells after treatment with different groups of exosomes. (**H**) Western blotting was performed to assess the expression of Collagen I, α-SMA, and FGFR3 protein in the different groups of exosome-treated NIH-3T3 cells. (**I**) The sequence of the miR-99a-5p target site in the 3′-untranslated region of *FGFR3*. (**J**) Interactions between *FGFR3* and miR-99a-5p in NIH-3T3 cells were verified using a dual-luciferase reporter assay. Data are presented as the means ± SD of values from experiments performed at least in triplicate. ** *p* < 0.01 vs. control group; ^#^ *p* < 0.05, ^##^ *p* < 0.01 vs. TGF-β1 group. n = 3.

Exosomes contain abundant miRNAs that are delivered to specific target cells, which is considered an important therapeutic effect [27]. By consulting the published literature [28], we found that some miRNAs, such as the let-7a and miR-99a-5p families, were highly enriched in the exosomes of MSCs, which were among the top-10 most highly expressed miRNAs (Figure 5A). Given the results of our bioinformatics analyses and the changes we observed in miR-99a-5p expression in pulmonary fibrosis, MSC-derived exosomes were added to NIH-3T3 cells. An obvious elevation in miR-99a-5p was observed in the NIH-3T3 cells (Figure 5B), indicating that the exosomes delivered miR-99a-5p to the recipient NIH-3T3 cells. To investigate the role of miR-99a-5p in exosomes, a miR-99a-5p mimic or inhibitor was used. Compared with the control group, the miR-99a-5p-specific mimic or inhibitor effectively elevated or reduced, respectively, the expression of miR-99a-5p in MSCs (Figure 5C). Corresponding changes were observed for exosomal miR-99a-5p extracted from MSCs culture media (Figure 5D). The in vitro experiments have shown that the levels of miR-99a-5p significantly decreased in TGF-β1-treated NIH-3T3 cells. Next, exosomes from the three groups were added to TGF-β1-treated NIH-3T3 cells, and the miR-99a-5p levels of cells were assessed. The results showed a decrease in miR-99a-5p levels after treatment with exosomes from MSCs transfected with miR-99a-5p inhibitors (Exo/miR-99a-5p inhibitor) and an increase in miR-99a-5p levels after treatment with exosomes from MSCs transfected with miR-99a-5p mimics (Exo/miR-99a-5p mimics), compared with the group not treated with MSC-exosomes (TGF-β1) (Figure 5E).

Subsequently, a series of functional tests were performed. MTS cell proliferation experiments showed that co-culture of TGF-β1-treated NIH-3T3 cells with MSC-exosomes significantly inhibited cell proliferation, and this inhibitory ability was enhanced after treatment with Exo/miR-99a-5p mimics. Further, this inhibitory effect was reversed after treatment with Exo/miR-99a-5p inhibitors (Figure 5F). These effects of MSC-exosomes were supported by the results of the transwell assays (Figure 5G). In TGF-β1-stimulated fibroblasts, α-SMA, Collagen I, and FGFR3 protein expressions were significantly reduced after Exo/miR-99a-5p mimic treatment. In contrast, no significant difference was observed after Exo/miR-99a-5p inhibitor treatment, suggesting that differences in the expression of these fibrotic markers may be the result of exosome-derived miR-99a-5p regulation (Figure 5H).

Next, wild-type and mutant *FGFR3* DNA segments containing the miR-99a-5p binding site were cloned into luciferase vectors (Figure 5I). The results of the luciferase assay showed a significant decrease in luciferase activity in NIH-3T3 cells co-transfected with the wild-type binding site vector in the presence of an miR-99a-5p mimic (Figure 5J), verifying that miR-99a-5p directly targets FGFR3 in NIH-3T3 cells.

### 2.6. FGFR3-Regulated TGF-β1-Induced Pulmonary Fibroblast Transdifferentiation Through the MAPK Signalling Pathway

**Figure 6:**
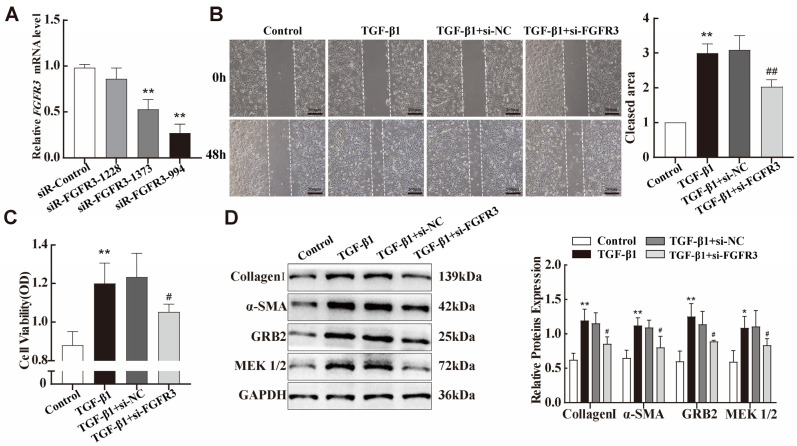
FGFR3 regulates TGF-β1-induced pulmonary fibroblast transdifferentiation through the MAPK signalling pathway. (**A**) qPCR was performed to determine the transfection efficiency of the three siR-FGFR3 species introduced into NIH-3T3 cells. (**B**,**C**) MTS cell proliferation and wound-healing assays were used to evaluate the viability and migration abilities of NIH-3T3 cells transfected with siR-FGFR3 or siR-NC before TGF-β1 treatment. (**D**) The expression levels of collagen-I, α-SMA, GRB2, and MEK1/2 in NIH-3T3 cells were assessed using Western blotting. Data are presented as the means ± SD of values from experiments performed at least in triplicate. * *p* < 0.05, ** *p*< 0.01 vs. control group; ^#^ *p* < 0.05, ^##^ *p*< 0.01 vs. TGF-β1 group. n = 3.

As mentioned before, FGFR3, a target of miR-99a-5p, showed increased expression both in vitro and in vivo silicosis models. Subsequently, we constructed siRNAs targeting FGFR3, transfected them into fibroblasts to exogenously inhibit the expression of *FGFR3* mRNA, and verified the transfection efficiency via qPCR (Figure 6A). The siR-FGFR3-994 was chosen to use for all subsequent experiments. MTS cell proliferation and migration assays were then used to detect the proliferative and migratory abilities of the fibroblasts. We observed that, by reducing FGFR3 expression, TGF-β1-treated fibroblast activation was suppressed (Figure 6B,C). Importantly, knockdown of FGFR3 attenuated TGF-β1-induced changes in the expression of fibrosis markers, including Collagen I and α-SMA (Figure 5D). The MAPK signalling pathway plays a critical role in governing diverse cell functions, including proliferation, differentiation, and apoptosis [29]. Through KEGG signalling-pathway analysis, we found that FGFR3 is an upstream signalling molecule for the MAPK pathway. Therefore, we hypothesised that FGFR3 regulates fibroblast proliferation via the MAPK signalling pathway. The expression of several key proteins in the MAPK signalling pathway was then examined via Western blotting. The results indicated that GRB2 and p-MEK1/2 expression was upregulated in TGF-β1-treated NIH-3T3 cells, which was reversed upon transfection with FGFR3-specific siRNA (Figure 5D).

### 2.7. miR-99a-5p Transmitted by MSC-Derived Exosomes Inhibited MAPK Pathway by Targetting FGFR3 in Mouse Lung Tissue of Silicotic Model

**Figure 7:**
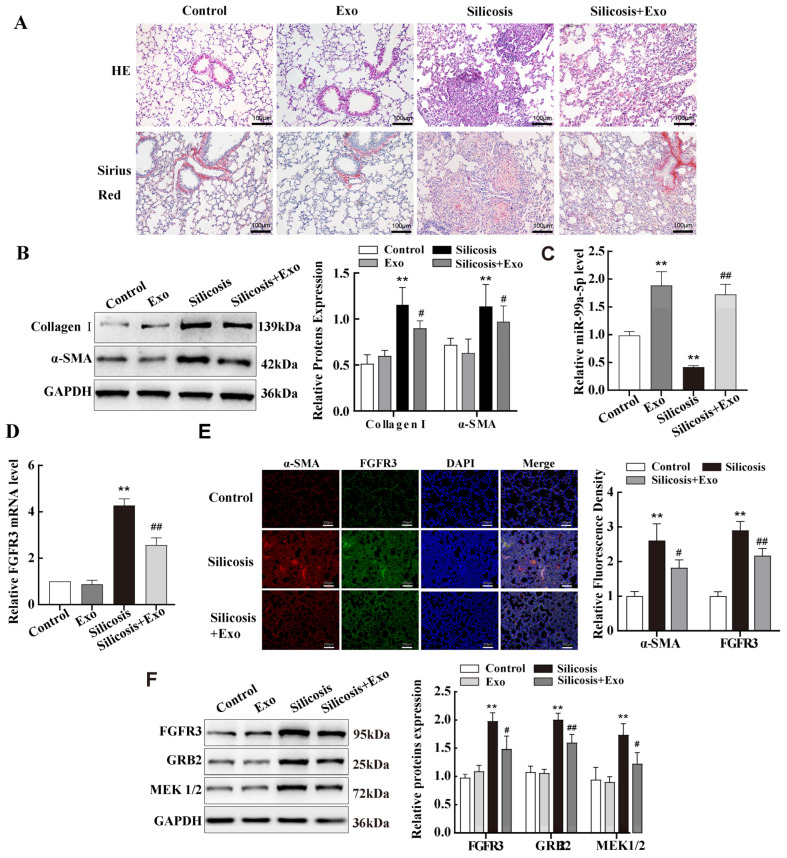
miR-99a-5p transmitted by MSC-derived exosomes inhibited MAPK pathway by targeting FGFR3 in mouse lung tissue of silicotic model. (**A**) Pathologic changes in mice were observed via haematoxylin and eosin (HE) and Sirius red staining. (**B**) Western blotting was used to examine the expression levels of Collagen I and α-SMA protein in a mouse model of silicosis. (**C**) qPCR results of miR-99a levels in a mouse model of silicosis after treatment with MSC-exosomes. (**D**) qPCR results of *FGFR3* mRNA levels in the mouse model. (**E**) Immunofluorescence assay results of FGFR3 and α-SMA protein in mouse lung tissues. (**F**) Western blotting was performed to examine the expression of FGFR3, GRB2, and MEK1/2 in the lung tissues of a mouse model. Data are presented as the means ± SD of values from experiments performed at least in triplicate. ** *p* < 0.01 vs. control group; ^#^ *p*< 0.05, ^##^ *p* < 0.01 vs. silicosis group. n = 6.

Firstly, to determine the therapeutic effect of MSCs exosomes on fibrosis in vivo, exosomes were injected into a silicosis mouse model via tail vein injection after 14 d of SiO_2_ exposure. The results showed that collagen fibres and silicosis nodules were significantly reduced after MSC-exosome treatment (Figure 7A). Western blotting results indicated decreased expression of α-SMA and Collagen I proteins (Figure 7B). Since FGFR3 is a target of miR-99a-5p, next, we explored the role of FGFR3 in lung fibrosis. We detected elevated miR-99a expression in the lung tissues of mice with silicosis after exosome treatment (Figure 7C). The qPCR results showed that FGFR3 expression was increased in the lung of mice with silicosis compared with those in the control group, and decreased after exosome treatment (Figure 7D). Immunofluorescence and Western blotting results confirmed these changes at the protein level, and these results were consistent with those from the cell model described above (Figure 7E,F). In addition, MAPK signalling-pathway-related proteins GRB2 and MEK1/2 showed the same trend in expression as FGFR3 in the lung tissues of the mouse model (Figure 7F).

These results suggest that FGFR3 regulates fibroblast activation through the MAPK signalling pathway. miR-99a was significantly downregulated and FGFR3 was upregulated in pulmonary fibrosis.

## 3. Discussion

Silicosis, commonly observed in mining and construction workers, is caused by inhaling dust with crystalline silica [30]. The incidence of silicosis is gradually increasing in response to the development of industries such as denim manufacturing and stone processing [5,31]. However, the current treatments for silicosis do not significantly improve survival, making the development of new treatments critical. In this study, we found that MSC-derived exosomes inhibited TGF-β1-induced fibroblast transdifferentiation and exerted protective effects in a mouse model of silicosis. Furthermore, we showed that exosomal miR-99a-5p regulated fibroblast activation through the FGFR3 and downstream MAPK signalling pathway, thereby alleviating the pathological progression of silicosis. This may provide new therapeutic strategies for the clinical management of silicosis.

MSCs are multipotent cells that can regenerate damaged tissue [32]. Several studies have shown that MSC-based stem cell therapy can alleviate the pathological effects of lung diseases, including pulmonary fibrosis [11,33]. However, the specific molecular mechanisms underlying these effects are not well defined.

The activation of pulmonary fibroblasts into myofibroblasts, which secrete large amounts of ECM and produce fibrosis-associated proteins, is thought to be critical in the pathology of pulmonary fibrosis [34,35]. We found that MSCs effectively inhibited the TGF-β1-induced transdifferentiation of fibroblasts and elevation of fibrosis markers, suggesting that MSCs have therapeutic potential for silicosis. Nevertheless, differences in the preparation, fitness, and function of MSCs hinder their application in clinical therapies [36]. Several studies have shown that MSCs exert their therapeutic effects by secreting exosomes [37,38]. Exosomes are natural nanocarriers that can deliver drugs, specific miRNAs, and proteins to their target cells [39]. Compared with stem cell therapies, exosomes are stable in body fluids and tissues and exhibit low immunogenicity and toxicity [40], offering the potential for broad clinical applications. In this study, we confirmed that MSC-exosomes can be taken up by fibroblasts and have therapeutic effects similar to those of MSCs in an in vitro cellular model. Importantly, multiple studies have found that MSC-exosomes have a tendency to home to wounded tissue [41,42]. An injection of exosomes into a mouse model of silicosis decreased the expression of pulmonary fibrosis marker proteins and effectively alleviated SiO_2_-induced collagen deposition in lung tissues, confirming the therapeutic efficacy of MSC-exosomes in silicosis. Notably, the amount and content of exosomes produced by MSCs may vary in different tissues or conditions, and there may be differences in the sensitivity of receptor cells to exosomes. Therefore, further studies are needed to explore the mechanisms underlying the optimisation of exosome treatment. Studies have demonstrated that aberrant miRNA expression is common in fibrotic diseases [43]. Moreover, exosomes have emerged as important participants in the pathological processes affecting fibrosis by transferring miRNAs to target cells [44]. In this study, we explored the effects of silicosis-associated exosomal miRNAs on fibrosis. Through bioinformatics analysis, we found that miR-99a-5p expression was decreased in an animal model of silicosis, which was subsequently validated via qPCR in in vivo and in vitro models of pulmonary fibrosis. In addition, Dinh et al. found that MSCs release miR-99a-5p-rich exosomes [28]. Therefore, we proposed that MSC-exosomes deliver miR-99a-5p to target cells to ameliorate silica-induced pulmonary fibrosis. This was validated in our study, as we observed a significant increase in miR-99a-5p levels in NIH-3T3 cells co-cultured with exosomes.

Several studies have indicated that decreased miR-99a-5p expression is strongly associated with various diseases [45,46]. In addition, Feliciano et al. showed that miR-99a-5p induces apoptosis and cell cycle arrest in lung cancer cells by regulating its target genes, leading to decreased proliferative capacity [47]. However, no reports pertaining to the association between miR-99a-5p and pulmonary fibrosis exist. In this study, we demonstrated for the first time that exosomes overexpressing miR-99a-5p inhibited TGF-β1-induced fibroblast activation compared with untransfected MSC-exosomes; this trend was reversed in exosomes with a low miR-99a-5p expression. These observations were confirmed by Western blotting and immunofluorescence analyses. Therefore, we proposed that the delivery of miR-99a-5p to lung fibroblasts represents an alternative mechanism for exosome-mediated pulmonary protection. The use of exosomes containing miR-99a-5p may be a promising approach for silicosis treatment.

FGFR3 is a member of the transmembrane receptor tyrosine kinase family [48]. Chakraborty et al. found that the upregulation of FGFR3 in systemic sclerosis promoted fibroblast activation and tissue fibrosis [49]. However, how FGFR3 functions in silicosis remains unclear. Here, we discovered that FGFR3 expression was significantly elevated in NIH-3T3 cells treated with TGF-β1, whereas exosomes overexpressing miR-99a-5p suppressed the expression of FGFR3. A dual-luciferase assay revealed that *FGFR3* is a target gene of miR-99a-5p, and KEGG analysis revealed that the predicted target genes of miR-99a-5p were enriched in the MAPK signalling pathway. In addition, studies have shown that FGFR3 plays a critical role in various diseases, including pulmonary fibrosis, by regulating the MAPK signalling pathway [50,51]. This is consistent with the results of our study, wherein the MAPK signalling pathway was activated in a silicosis mouse model and this effect was reversed by exosome treatment. Importantly, knockdown of FGFR3 inhibited activation of the MAPK signalling pathway and decreased expression of the fibrosis markers α-SMA and Collagen-I, which was caused, at least in part, by inhibiting fibroblast activation. Moreover, we demonstrated that FGFR3 directly interacted with GRB2 to regulate the MAPK signalling pathway.

In summary, our results suggest that MSC-derived exosomes attenuate SiO_2_-induced pulmonary fibrosis. Importantly, MSC-derived exosomes deliver miR-99a-5p to fibroblasts and inhibit fibroblast transdifferentiation by targeting FGFR3 and regulating MAPK signalling, which indicates that they could be promising candidates for the development of silicosis therapies. However, exosomes are enriched with other miRNAs, proteins, and mRNAs, and further investigation is required to determine how other exosome components are involved in this process. In addition, further experiments on the applicability of our findings to a therapeutic setting are limited by the lack of adequate clinical data. Particularly, exosomes can be taken up by multiple types of recipient cells, including fibroblasts, via patterns of receptor–ligand binding, endocytosis, or membrane fusion [52]. Finding more effective methods to target lung fibroblasts in vivo will be the focus of future efforts.

## 4. Materials and Methods

### 4.1. Cell Culture and Reagents

NIH-3T3 cells and MSCs (they were isolated from epididymal white adipose of 6-week-old C57BL/6J mice and immortalised by transformation with SV40 virus large T antigen) were purchased from the AnWei-SCI company (Shanghai, China). NIH-3T3 cells were cultivated in Dulbecco’s modified Eagle’s medium (Gibco, Brooklyn, NY, USA) supplemented with 10% fetal bovine serum (Gibco, Brooklyn, NY, USA) and maintained at 5% CO_2_ and 37 °C. NIH-3T3 cells were treated with different concentrations of human recombinant TGF-β1(PeproTech, Cranbury, NJ, USA) to construct cell models followed by MSC-derived exosome treating (final concentration: 50 μg/mL).

### 4.2. Co-Culture Model of TGF-β1-Treated NIH-3T3 Cells with MSCs

Six-well plates were inserted into a transwell device with a 4 μm porous polycarbonate membrance (Corning Inc., Corning, NY, USA). TGF-β1-treated NIH-3T3 cells were cultured in the six-well plate, while MSCs were cultured in the upper chamber of the transwell, and NIH-3T3 cells were collected 24 h later for analysis.

### 4.3. Mouse Model of Silicosis and Group

C57BL/6J mice were randomly divided into six groups of eight mice each, including the control group (0-day), the 7-, 14-, and 28-day groups, exosome control group, and the exosome treatment group. The silicosis mouse group was administered a silica suspension (Sigma Aldrich, St. Louis, MO, USA; 10 mg to each mouse; 0.05 mL) by intratracheal instillation, and the control group was without any treatment. The exosome treatment group was injected with 200 µg/100 µL(PBS) MSC-exosomes in the tail vein every 4 d, commencing 14 d after silica treatment [18]. The animal study protocol was approved by the Ethics Committee of the North China University of Science and Technology (protocol code: 2023-033; date of approval: 2 July 2023) for studies involving animals.

### 4.4. Isolation, Characterisation, and NIH-3T3 Uptake of Exosomes

MSCs were cultured in exosome-free serum for 48 h, and the spent medium was collected and centrifuged sequentially at 300× *g* for 10 min, 2000× *g* for 10 min, and 10,000× *g* for 1 h to remove the cell fragments. Finally, the supernatant was centrifuged at 100,000× *g* for 2 h. Next, the supernatant was resuspended in PBS, filtered using a 0.22 μm filter (Biofil, Guangzhou, China), and centrifuged again at 100,000× *g* for 2 h. Finally, the resulting precipitate was resuspended in PBS and used for subsequent experiments. Exosomes were quantified by measuring exosome protein concentration using BCA kit (Thermo Fisher, Waltham, MA, USA).

Prior to use, the particle size of the isolated exosomes was examined using a nanoparticle size analyser (Nano-ZS90, Malvern, UK); the exosome markers CD63 and TSG101 (GeneTex, Irvine, CA, USA) were detected via immunoblotting, and their morphology was observed using transmission electron microscopy (Hitachi, Tokyo, Japan). For exosome tracing, green fluorescent dye DiO-labelled (Beyotime, Shanghai, China) exosomes were added to NIH-3T3 cells, the cytoskeleton was stained with TRITC-phalloidin (Beyotime, Shanghai, China) for 4 h, and the nuclei were stained with DAPI (Abcam, Boston, MA, USA). Finally, the cells were visualised using confocal laser scanning microscopy(Olympus, Tokyo, Japan).

### 4.5. Histopathology (HE and Sirius Red Staining)

For histopathology, lung tissues were immediately removed, fixed in 4% paraformaldehyde (Biosharp, Beijing, China) for 48 h, dehydrated in gradient ethanol, and embedded in paraffin. The resulting tissue blocks were cut into 4 µm-thick sections. The sections were stained with haematoxylin and eosin for pathological analysis. Morphological changes were assessed for collagen fibre distribution by staining with Sirius red and subsequently observed under a microscope.

### 4.6. Cell Proliferation and Migration

Cell proliferation was determined using the MTS assay (Promega, Madison, WI, USA). Briefly, 5 × 10^3^ NIH-3T3 cells were plated in 96-well culture plates. The cells were treated with 100 μL exosomes after TGF-β1 stimulation. After 48 h, 20 μL of MTS reagent was added to each well, and the cells were incubated at 37 °C for another 2 h. Optical densities at 490 nm were measured using a SpectraMax M5 microplate reader (Molecular Devices, San Jose, CA, USA).

Cell migration was assessed using a wound-healing assay. The NIH-3T3 cells (5 × 10^5^/well) were seeded into 6-well plates. The wounds were formed by scratching the cell monolayer with a plastic tip along a straight line, and wound closure was observed after 48 h. Photographs of cells before and after migration were taken under a microscope (Olympus, Tokyo, Japan), and the area recovered was calculated.

### 4.7. Cell Transfection

Small interfering RNA (siRNA) and miRNA mimics or inhibitors were purchased from GenePharma (Shanghai, China). Their sequences are shown in Table S1. NIH-3T3 cells were seeded in 6-well plates, and when cell confluency reached 80%, 50 nM siR-FGFR3 or 30 nM miR-99a mimics/inhibitors were transfected into the cells using a HighGene transfection reagent (ABclonal, Wuhan, China).

### 4.8. Western Blot

Total proteins from mouse lung tissues and cells were extracted using RIPA buffer (Beyotime) supplemented with 1% protease inhibitor, and protein concentration was measured using a BCA protein assay. A total of 20 µg protein was separated via 10% SDS-PAGE. The resolved proteins were transferred onto nitrocellulose membranes, which were then blocked with 5% non-fat milk at room temperature (RT) for 2 h. The membranes were incubated overnight at 4 °C with the following specific primary antibodies: FGFR3, GRB2, MEK1/2, collagen I, α-SMA, Beclin1, LC3α/β, and phospho-mTOR (1:1000, Affinity, Cincinnati, OH, USA). After washing with tris-buffered saline with 0.1% Tween^®^ 20 Detergent (TBST), the membranes were incubated with the goat anti-rabbit IgG or goat anti-mouse IgG secondary antibody (Affinity) for 2 h at RT. The intensity of protein bands was measured using an ECL detection reagent (Applygen, Beijing, China) and analysed using the ImageJ software, version1.53t (NIH, Washington, DC, USA).

### 4.9. RNA Extraction and Quantification

Total RNA was extracted from cells and lung tissues using TRIzol reagent (Invitrogen, Waltham, MA, USA). Reverse transcription was performed using a PrimeScript™ RT reagent kit (Takara, Shiga, Japan), and, for real-time PCR, the TB Green^®^ Premix Ex Taq™ II (Takara, Shiga, Japan) protocol was instituted on an Applied Biosystems 7500 Real-Time PCR System (Thermo, Waltham, MA, USA). Relative mRNA expression was calculated using the 2^−ΔΔCT^ method. Primers were purchased from GenePharma (Shanghai, China), and their sequences are listed in Table S2.

### 4.10. Immunofluorescence Assays

NIH-3T3 cells were fixed with 4% paraformaldehyde for 30 min and blocked with goat serum for 20 min at RT. Next, the cells were incubated overnight at 4 °C with mouse anti-α-SMA and rabbit anti-FGFR3 antibodies (1:200, Affinity). Lung tissue sections were dewaxed with xylene and dehydrated using an ethanol gradient. The subsequent steps were the same as those used for immunohistochemistry. Next, the cells or tissue sections were washed thrice with phosphate-buffered saline and incubated with fluorescent goat anti-mouse IgG or donkey anti-rabbit IgG secondary antibody (Invitrogen, Carlsbad, CA, USA) at 37 °C for 1.5 h. Nuclei were stained with DAPI (Abcam, Cambridgeshire, UK) and observed under a fluorescence microscope (Olympus, Tokyo, Japan).

### 4.11. Dual-Luciferase Reporter Assay

Reporter assays were used to examine the relationship between miR-99a and FGFR3 expression. We cloned oligos containing the predicted miR-99a binding site in the FGFR3 3′-untranslated region and its corresponding mutant form into a psiCHECK-2 vector (GenePharma, Shanghai, China). HighGene transfection reagent (ABclonal, Wuhan, China) was used to co-transfect FGFR3-wt or FGFR3-mut vectors with miR-99a mimics or mimic controls into NIH-3T3 cells. After 48 h of transfection, firefly luciferase activity was measured using a dual-luciferase assay system Promega (Marin, WI, USA) and adjusted using Renilla fluorescence.

### 4.12. Statistical Analysis

Data analyses were performed using SPSS 23.0 software. All data are expressed as means ± standard deviation (SD). Comparisons between two groups were performed using independent sample *t*-tests, while those between multiple groups were performed using a one-way analysis of variance (ANOVA). Differences were considered significant when the two-tailed *p*-values were <0.05.

## 5. Conclusions

The results above indicate that pulmonary fibroblasts transdifferentiate promotes the development of SiO_2_-induced pulmonary fibrosis. MSC-derived exosomes alleviate pulmonary fibrosis in a silicosis model. Particularly, MSC-derived exosomes deliver miR-99a-5p to fibroblasts and inhibit fibroblast activation by suppressing FGFR3 and downstream MAPK signalling pathway, which indicates a potentially effective treatment for silicosis.

## Data Availability

The original contributions presented in the study are included in the article and Supplementary Materials, further inquiries can be directed to the corresponding author.

## Supplementary Materials

The following supporting information can be downloaded at: https://www.mdpi.com/article/10.3390/ijms252312626/s1.
