# Supplementary material for: Mesenchymal Stem Cell-Exosomal miR-99a Attenuate Silica-Induced Lung Fibrosis by Inhibiting Pulmonary Fibroblast Transdifferentiation
Source: Int J Mol Sci. 2024 Nov 25;25(23):12626. doi: 10.3390/ijms252312626 (PMC11641662; doi:10.3390/ijms252312626)
Supplement: Supplementary file 1 [file ijms-25-12626-s001.zip › ijms-3266356-supplementary.pdf]

Supplementary information.

Table S1. The sequences of miR-99a mimic/inhibitor and siR-FGFR3

Primer	Sequence
Mmu-miR-99a mimic	sense (5′-3′)-AACCCGUAGAUCCGAUCUUGUG
	antisence (5′-3′)-CAAGAUCGGAUCUACGGGUUUU
Mmu-miR-99a inhibitor	sense (5′-3′)-CACAAGAUCGGAUCUACGGGUU
siR-FGFR3-1228	sense (5′-3′)-GCGCUAACACCACCGACAATT
	antisence (5′-3′)-UUGUCGGUGGUGUUAGCGCTT
siR-FGFR3-1373	sense (5′-3′)-GGAGCUGAUGGAAACUGAUTT
	antisence (5′-3′)-AUCAGUUUCCAUCAGCUCCTT
siR-FGFR3-994	sense (5′-3′)-GCAGCAUCCGGCAGACAUATT
	antisence (5′-3′)-UAUGUCUGCCGGAUGCUGCTT

Table S2. The sequences of primer pairs

Primer	Sequence	Amplification
miR-99a	Forward 5′-AATGCTCAAACCCGTAGATCC-3′	78
	Reverse 5′-TATGGTTGTTCTGCTCTCTGTCTC-3′	
U6	Forward 5′-CGCTTCGGCAGCACATATAC-3′	87
	Reverse 5′-TTCACGAATTTGCGTGTCATC-3′	
FGFR3	Forward 5′-CGACAGGTGTCCTTGGAATC′	168
	Reverse 5′-GACATGGGGTTCTTGGACTGT-3′	
Collagen I	Forward 5′-TGCATGTCCGATGTTTCCAG′	21
	Reverse 5′-CTTACCAAGTGTCAGCCGGG-3′	
GAPDH	Forward 5′-CATCACTGCCACCCAGAAGACTG-3′	141
	Reverse 5′-ATGCCAGTGAGCTTCCCGTCAG-3′	

Figure S1

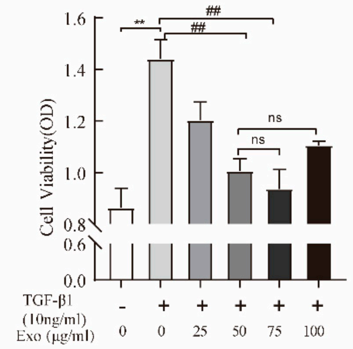

Figure S1. MTS assay was performed to optimize the treatment concentration of MSC-exosomes to inhibit the proliferation of NIH-3T3 cells induced by 10 ng/ml TGF-β1. ** $P < 0.01$ vs. control group; ## $P < 0.01$ vs. TGF-β1 group, n = 3.
